# CREATING AN OBESITY BILL OF RIGHTS–INFORMED CLINICAL PRACTICE: A GERIATRICIAN’S PERSPECTIVE

**DOI:** 10.1093/geroni/igae098.1705

**Published:** 2024-12-31

**Authors:** John Batsis

**Affiliations:** University of North Carolina, Chapel Hill, North Carolina, United States

## Abstract

The Obesity Bill of Rights (OBOR) sheds light on many challenges and opportunities for clinicians to implement in their respective practices. Dr. Batsis will share themes and insights that emerged from recent obesity roundtable discussions hosted by the National Council on Aging and leading up to the creation of the OBOR, including special considerations for obesity prevention and care for older adults aged 65 and over from a clinical standpoint. Barriers to obesity treatment; health care access; and access to trustworthy information for older adults—including those that intersect with issues of diversity, equity, and inclusion—will be explored. Examples of steps Dr. Batsis has taken to align his own delivery of care with the OBOR will be highlighted, as well as recommendations to facilitate open and responsive dialogue built on trust between patients and providers.

